# Virus encoded circulatory miRNAs for early detection of prostate cancer

**DOI:** 10.1186/s12894-015-0111-9

**Published:** 2015-11-26

**Authors:** Jayoung Kim, Seok Joong Yun, Wun-Jae Kim

**Affiliations:** Departments of Surgery and Biomedical Sciences, Cedars-Sinai Medical Center, 8700 Beverly Blvd., Davis Room 5071, Los Angeles, CA 90048 USA; Departments of Medicine, University of California, Los Angeles, CA USA; Department of Surgery, Harvard Medical School, Boston, MA USA; Department of Urology, Chungbuk National University College of Medicine, 62 Kaeshin-dong, Heungduk-gu, Cheongju 361-763 Republic of Korea

**Keywords:** miRNAs, Virus, Extracellular vesicles, Prostate cancer

## Abstract

**Background:**

Prostate cancer (PCa) is the most commonly diagnosed cancer and kills about 28,000 American men annually. Although progress has been made in understanding the molecular features of different forms of the disease, PCa is considered incurable when it becomes resistant to standard therapies. Prostate specific antigen (PSA) test has been a gold standard of diagnosis for PCa, however, it can result in lead to the unnecessary biopsies and treatment of indolent cancers due to the low specificity. Thus, the limitations of PSA screening for PCa have prompted much focus on strategies how to enhance the accuracy of PSA for distinction between aggressive and indolent cancers.

**Discussion:**

Studies of miRNAs in PCa patients have suggested differentially expressed miRNAs between healthy controls and those with PCa, providing potential biomarker candidates using body fluids including urine and blood. Virus infection has been considered to associate with PCa incidence. Virus infected PCa cells may shed extracellular vesicles and communicate with neighboring cells, which were not infected yet, however, no mechanistic approaches were performed to understand the biology. The miRNAs composition in the shedding extracellular vesicles, and its role in PCa are completely undefined. In the near future, new insights to connect between the viral derived miRNAs and PCa progression might provide an opportunity to diagnose, risk prediction and therapeutic strategies.

**Summary:**

The goal of this debate article is to provide a short review on miRNAs, virus infection and viral encoded miRNAs in PCa, with a primary focus on circulating miRNAs as potential non-invasive biomarkers for PCa patients.

## Background

Prostate cancer (PCa) remains one of the most commonly diagnosed malignant tumor in men and the second leading cause of death from cancer [1–3]. Several new drugs including Radium-223, Cabazitaxel, Sipuleucel-T, Abiraterone, and Enzalutamide have shown significantly improved survival in castration-resistant metastatic disease (CRPC) patients in Phase 3 trials [4–8]. For early detection of PCa, urologists rely on serum prostate-specific antigen (PSA) testing or digital rectal examination (DRE) [9]. Serum PSA testing has successfully achieved a dramatic increase of PCa detection, however, PSA testing has a low specificity because an increase of PSA level is not a PCa-specific event. Serum PSA levels are also elevated in men with benign prostatic hyperplasia and prostatitis. Limitations of DRE for the early detection derived from its low accuracy and the dependence on highly trained clinicians. Such a lack of PCa-specific early detection tool leads to create unnecessary biopsies or severe treatments for indolent PCa [2]. Development of methods of prebiopsy risk stratification and more simple, noninvasive, sufficiently sensitive and specific tests for PCa diagnosis would allow the stratification of PCa patients who are at the very early stage of disease [1, 10]. Current efforts to improve the accuracy of PSA and develop new biomarkers for PCa may hold the promise of improving the screening, diagnosis, and monitoring of prostate cancer. PCA3, a non-coding and large chain RNA that is significantly overexpressed in PCa compared to non-tumorous prostate cells, was introduced as a biomarker for PCa to show high sensitivity (52 % to 58 %) and specificity (72 % to 87 %) [11–16]. The US FDA approved PCA3 as a risk assessment tool for PCa to guide prostatic biopsy among men with negative previous prostate biopsies. Urinary PCA3 assay combined with TMPRSS2:ERG is reported to improve the diagnostic accuracy [17, 18]. Recently reported studies suggested that these requirements could be fulfilled using the diagnostic approach based on analysis of urine, which has become the future for non-invasive biomarker testing [10, 19].

This brief debate article is to discuss ad seek to characterize the circulating microRNAs (miRNAs) in urine with regard to PCa. We aim to provide our audience the current knowledge, in particular, focused on clinical implication as a liquid biopsy with a clinically satisfactory degree of sensitivity and specificity.

## Discussion

### MicroRNAs in prostate cancer

#### What are microRNAs?

Small non-coding microRNA (miRNA) molecules are known as post-transcriptional regulators involved in the regulation of gene and protein expression by interfering in the post-transcriptional level, resulting in the degradation and translation inhibition of mRNAs. Approximately more than 2,000 mature human miRNAs have been reported so far. These miRNAs are short (19–24 nt) and low molecular weight RNAs. miRNAs are derived from hairpin-like precursor transcripts (pre-miRNAs) and taken out of nucleus by a mediator protein, exportin 5. Pre-miRNA is then cleaved by Dicer (a ribonuclease III enzyme) to excise the mature miRNAs in the form of siRNA-like duplexes and asymmetrical assembling of the mature miRNA strands. Since miRNAs interact with multiple messenger RNAs by binding the pairing of bases of the mRNAs and repress target gene expression, and regulate mRNA cleavage and mRNA decay initiated by the miRNA-guided rapid deadenylation, miRNAs have a wide variety of functions contributing to various pathological conditions including prostate cancer [20].

### How miRNAs are connected with PCa development and progression cancer?

**Fig. 1:**
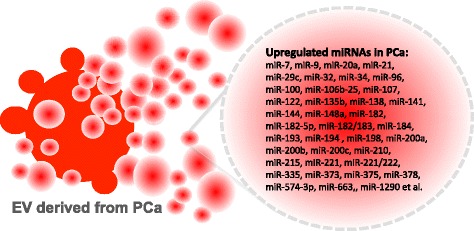
miRNAs upregulated in PCa

Many of miRNAs play roles in cell proliferation and apoptosis processes, thus miRNA expression profiles can be considered as useful biomarkers monitoring many types of cancer progression and treatment responses [21]. Previous studies have suggested several miRNA biomarker candidates associated with PCa [22]. miR-548c-3p was found significantly overexpressed in CRPC. Expression levels of miR-548c-3p negatively correlated with recurrence-free survival. miR-375 was significantly downregulated in 83.5 % of PC patients compared to BPH controls. Metastatic CRPC patients with chemotherapy-resistant had the higher miR-21 levels compared with those with hormone dependent primary PCa. A combined diagnostic testing of miR-21 and PSA could stratify patients effectively. miR-21 was introduced as one of miRNAs of a multiple diagnostic profile, which includes miR-21,miR-141, and miR-221. miR-141 was independently identified as a biomarker of CRPC. Another combination panel of PSA and expression levels of let-7c, miR-30c, miR-141, and miR-375 was suggested as diagnostic biomarkers for PCa screening outperforming the PSA testing alone. Some PCa-specific miRNAs were identified from mouse PCa model. A set of 46 miRNAs in the serum of transgenic mice with advanced adenocarcinoma PCa were identified. Among those, miR-141, miR-298, and miR-375 were found to be elevated in the serum of metastatic CRPC patients. In particular, miR-141 and miR-375 showed a correlation with disease outcome. Diagrammatic representation of miRNAs associated with PCa is shown in Fig. 1.

### What can be the target genes or pathways of miRNAs in PCa?

In the accumulated literature the roles of miRNAs in the pathobiology of PCa were found in cell cycle, apoptosis, epithelial to mesenchymal transition (EMT) and mesenchymal to epithelial transition (MET) states, invasion and metastasis, PCa stem cell, and androgen receptor (AR) pathway. PTEN-PI3 Kinase-AKT, EGFR and AR pathways are considered as the most important signaling pathways in PCa. Upregulation of miR-221/-222 in PCa suggest a mechanistic link to PCa since miR-221/-222 plays a role in AR pathway regulation, c-kit, PTEN, and TIMP3 et al. miR-124 was characterized to have a direct targeting of AR thereby inducing down-regulation of miR-125b and up-regulation of p53. When miR-125b is suppressed miR-125b effectors (p52, Puma, Bak1, and p14ARF) were downregulated. The regulation of miR-125b on apoptotic proteins (mitochondrial cytochrome C and Caspase-3) and NCOR2 (a co-repressor of AR) was well known. Another PCa-associated miRNA, miR-let-7c regulates AR pathway and involves in the conversion of hormone dependent PCa to CRPC.

### Urinary microRNAs as prostate cancer biomarkers

Several cancer-associated miRNAs were found in circulating body fluids such as urine, which make it possible to develop non-invasive biomarkers mainly due to their ease of access and stability. Only few studies for PCa-associated miRNA in urine were reported. Five of the miRNAs that were differentially quantified in PCa patients compared to controls (miR-107, miR-574-3p, miR375, miR200b and miR-141) were successfully quantified in urine of men with cancer, which were much higher in PCa patients than that of healthy volunteers. Such reports provide evidence that circulating miRNAs might be a next-generation biomarker and contribute to cancer screening in non-invasive liquid biopsy. Two additional promising miRNAs, miR-141 and miR-375 were found in the patient blood. In particular, data from this study showed that metastatic PCa patients have approximately 50 fold higher miR-141 levels, compared to the healthy individuals.

### Virus infection and prostate cancer

Among the various viruses, herpes virus is one of the viruses most commonly related to carcinogenesis. Several epidemiological studies evaluated the association between herpes virus infection and prostate cancer risk, although results were inconsistent. Herpes virus plays an important role in the pathogenesis of cancer via the inhibition of cell apoptosis and stimulation of DNA synthesis, which may ultimately lead to PC. Previous meta-analysis indicated that infection by herpes simplex virus type 2 (HSV-2) or human herpesvirus 8 (HHV-8) may be associated with a higher prostate cancer risk. However, there has not been a study to elucidate the potential mechanism of HSV-2 infection underlying viral PC carcinogenesis. A potential link between HPV infection and PCa risk was attempted to study the causal role of HPV-16 in prostate carcinogens. A significant increase of PCa risk related with HPV-16 infection was observed. However, this is still contradictory since other epidemiological studies could not find same association [23–25]. Therefore, an elaborate and comprehensive demonstration of the association between herpes virus infection and prostate cancer risk is of significance [24]. Further investigations and large-sample studies are required to and the relationship between herpes virus infection and prostate cancer risk.

**Fig. 2:**
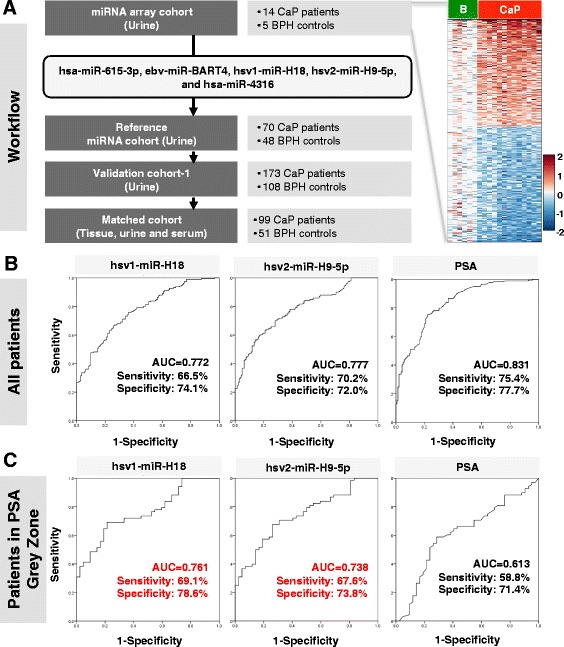
Diagnostic performance of urinary hsv1-miR-H18 and hsv2-miR-H9-5p compared with serum PSA levels (published data) [19]. **a** Overview of the study design. A heatmap representing miRNA microarray data suggested that miRNA signature segregates PCa from BPH controls (right). **b** ROC curves for all patients. **c** ROC curves for patients only within the PSA grey zone (3–10 ng/ml)

Our recent findings demonstrated the overexpression of herpes virus-encoded miRNAs in urine samples from prostate cancer patients, compared to those of control subjects [19]. Interestingly, hsv1-miR-H18 and hsv2-miR-H9-5p detected in urine samples showed better diagnostic performance than tPSA levels in patients within the PSA grey zone. A recent meta-analysis showed that HSV infection is associated with an increased PCa risk, however, it remains puzzling how these two particular vir-miRNAs contribute to PCa. To better understand the biological contribution of hsv1-miR-H18 and hsv2-miR-H9-5p to PCa, the geographic distribution, gender difference and socioeconomic variation of virus infection and their possible impact on prostate cancer should be considered to investigate (Fig. 2).

### Viral miRNAs circulating through biofluids

Viruses also encode their own sets of miRNAs, which they use to control the expression of either the host's genes and/or their own. In the past few years’ evidence of the presence of cellular miRNAs in extracellular human body fluids such as serum, plasma, saliva, and urine has been accumulated [26, 27]. Furthermore, it has been also demonstrated that miRNAs secreted by virus-infected cells are transferred to and act in uninfected recipient cells [19].

The first viral-encoded miRNAs was found in cells infected with EBV [27, 28]. The majority of natural viruses found to encode miRNAs. In DNA viruses (such as herpes virus, polyomavirus, ascovirus, baculovirus, iridovirus, and adenovirus families), a DNA component to replication cycle can exploit the initiating host miRNA biogenesis machinery in the nucleus, where they replicate and cause long-term persistent infections. Among DNA viruses, which account for the majority of known virus-encoded miRNAs, 95 % of viral miRNAs known today are of herpes virus origin. However, little has been identified about the role of circulating miRNAs from the virus-infected cells.

### Extracellular vesicles and their biological role of EV in prostate cancer

Cells can release different types of vesicles, which have been difficult to categorize in a definitive manner. The transmission of vesicles from cancer cells to other cell types has been the subject of intensive studies in recent years. It is a process that can make it possible a sophisticated form of cellular communication through the delivery of highly complex and dynamic cargo. As a delivery vehicle between cells, extracellular vesicles (EV) have been considered as a molecular cargo mediating a communication between cells in the microenvironment. Although EV were originally considered to be a means for exclusion of garbage molecules from cells, it is now clear that EV alter signaling pathways and have a biological influence in neighboring cells [29]. Also, since EV have the bioactivity of their molecular cargo and can be readily isolated from multiple biological fluids (e.g., urine, serum, plasma, pleural effusion, saliva), EV has been considered as a non-invasive biomarker candidate [30–32].

There are multiple types of EV, the small sealed membrane vesicles that are produced from cells differing in size, biology, contents, and secretory mechanisms. Exosomes and microparticles (MPs) are two distinct groups of EV. Exosomes are originated from the inward budding of the limiting membrane of multivesicular bodies (MVBs), while MPs are in bigger size vesicles formed by the outward membrane budding. Vesicles that are produced from cells during cell death are apoptotic bodies.

It is known that PCa patients had higher levels of urinary exosomes than the healthy donors. The membranes of exosomes are resistant to the osmeolytic and proteolytic activity of urine, indicating that exosomes are quite stable in urine. PC cell-derived EV can deliver various genetic factors such as nucleic acids, including DNA, mRNA, miRNA, and small non-coding RNAs, as well as oncoproteins and metabolites, leading to the horizontal transfer of oncogenic information to neighboring immune cells, vessel cells, or cancer cells [33, 34]. EV-mediated RNA transfer provides benefits as a communicasome [35–37]. However, little is known how EV-mediated transfer of these molecules enters into recipient cells, and how specific miRNA species can be sorted into EV. Our series of publications in this field suggest that EV shedding can influence on microenvironment of PCa by modulating immune cell proliferation and activation [38].

### EV influence immune response in prostate cancer

Role of immune response caused/mediated by miRNA was suggested by a series of studies showing that EV-derived miRNAs participate in the regulation of inflammatory responses. A recent report from our group demonstrated that EV derived from amoeboid phenotype of prostate cell may influence the immune response of the tumor microenvironment. A tritiated thymidine (^3^H-thymidine) incorporation assay revealed that EV contain miRNAs (e.g., miR-125a), which are transferred to tumor microenvironment leading to proliferative inhibition of immune cells. Other interesting findings suggest immune response may be mediated by miRNAs regulating Toll-like receptors (TLRs), which play an important role in immune response and inflammation including tissue repair and tissue injury-induced inflammation. In PCa cell lines, several miRNAs (e.g., has-miR-29b, −29c, −148b, and −152) were upregulated by TLR3 activation, leading to antitumoral effects on PCa. EV derived from dendritic cells contain miR-155 (a promoter of inflammatory responses) and miR-146a (a mediator of immune suppression), which are known as important players to regulate inflammation, alter the gene expression of inflammation related targets and reprogramm the response to endotoxin [39, 40].

### EV released from virus-infected cells

In virology field, biology of extracellular vesicles are well understood compared to cancer mainly because virus should be enveloped to be released from infected host. The accumulating evidence suggests that viruses, such as retroviruses, hepatitis C virus, herpes simplex virus, Epstein-Barr virus, Coxsackie virus B3 (CVB3), utilize the cellular vesiculation pathway for budding and assembly, immune evasion, and intercellular communication. It is also well known that many virally infected cells secrete vesicles (most of cases, exosomes) containing various viral proteins and genetic materials such as RNAs. Studies on herpes simplex virus-infected cells demonstrated that virus communicate via vesicles. In the case of Epstein-Barr virus, recent reports suggested that herpes virus utilizes exosomes, one kind of extracellular vesicles, as a mechanism of cell-to-cell communication and transfers signaling competent proteins and functional miRNAs to uninfected neighbor cells. For example, exosomes released from infected cells have been shown to contain co-receptors for HIV, which can enhance virus entry into cells. Proteins in the secreted exosomes from virus-infected cells can induce apoptosis in CD4 T cells, and contribute innate immune response.

### EV as cargoes to deliver viral miRNAs

EV-mediated communication would allow the virus to respond to the cellular microenvironment. Virus-infected cells continuously shed and transfer EV to uninfected neighboring cells. Throughout EV shedding and secretion to extracellular space, the virus-encoded miRNAs were delivered to other cells, leading to alteration of miRNA-mediated gene repression and intercellular communication. Thus, the presence of viral-encoded miRNAs in EV suggests that virus-infected cells perturb gene expression in the surrounding tissue, resulting in destruction of the immune system. Exosomes secreted from HIV-infected alveolar macrophages have been identified to carry viral miRNA (vir-miRNA) such as vmiR88, vmiR99, and vmiR-TAR. It has not been clearly identified how vir-miRNA composition is decided and recruited to EV. Vir-miRNAs might have a role in cancer initiation by blocking of major tumor suppressors (e.g., p53) or acceleration of cancer development by evading cellular immune response (e.g., mi17-92).

These findings still raise a number of exciting questions. Does virus-infected prostate cells secrete miRNAs using EV as a cargo? What are the target genes of the abundantly secreted viral miRNAs? Are extracellular vesicles shed only in the absence of viral replication? Is there any evidence for functional miRNA delivery in vivo? If so, what’s the biological mechanism? In addition, the detail mechanism is currently lacking whether virus infection promotes miRNAs sorting into EV, or whether this secretion is a selective and specific process.

## Conclusions

In PCa biomarker development, the greatest unmet need remains: a biomarker that stratifies men at risk of aggressive PCa or a biomarker that identify the early stage of patients who need active surveillance, eventually leading to a reduction of unnecessary interventions. Although miRNAs might have many useful clinical applications for patients with PCa, many additional studies are warranted to clarify their function and regulation during tumorigenesis and tumor progression. The studies to determine the role of circulating miRNAs during PCa progression would have the potential that PCa patients can be molecularly stratified based on their miRNAs profile in urine samples. In addition, the studies could uncover important clues about underlying disease mechanisms.

Urine analyses have great potential to be adapted in clinical practice, based on its non-invasiveness. Identification of non-invasive clinical indicators of PCa would be one of the most important advances achievable in this field. Our current mechanistic understanding of shedding vesicle biology and function, especially in the context of virus infection and communication with host, is not mature enough. Identification of potential candidates of the circulating miRNAs signature in patient urine samples would have significant implications for an alternative and/or supportive diagnostic tool for PCa.

Unnecessary diagnostic procedures would be minimized for patients with early stage of PCa, contributing to the easier diagnostic assessment and to the reducing associated public health and economic burden to patients. In addition, the studies to understand role and biology of the circulating miRNAs have significant clinical relevance to public health since it will improve accuracy of predicting patient survival, identifying responsiveness candidates of PCa patients. Furthermore, we envision that this study will also provide the mechanistic data to address the long-term goal of the PCa field identifying new treatments tailoring more specific and effective therapies for PCa, thus it holds great promise for the treatment of high risk PCa patients.

## Abbreviations

PCa: Prostate cancer
CRPC: Castration-resistant metastatic disease
PSA: Prostate-specific antigen
DRE: Digital rectal examination
miRNAs: MicroRNAs
EMT: Mesenchymal transition
MET: Mesenchymal to epithelial transition
AR: Androgen receptor
HSV-2: Herpes simplex virus type 2
HHV-8: Human herpesvirus 8
EV: Extracellular vesicles
MPs: Microparticles
MVBs: Multivesicular bodies
CVB3: Coxsackie virus B3
